# Learning From Countries on Measuring and Defining Community-Based Resilience in Health Systems: Voices From Nepal, Sierra Leone, Liberia, and Ethiopia

**DOI:** 10.34172/ijhpm.7996

**Published:** 2024-09-07

**Authors:** Angeli Rawat, Katrina Hsu, Agazi Ameha, Asha Pun, Kebir Hassen, Aline Simen-Kapeu, Nuzhat Rafique, Macoura Oulare, Jonas Karlstrom, Sameera Hussain, Kumanan Rasanathan

**Affiliations:** ^1^School of Population and Public Health, University of British Columbia, Vancouver, BC, Canada.; ^2^Faculty of Medicine, University of Alberta, Edmonton, AB, Canada.; ^3^UNICEF Ethiopia Country Office, Addis Ababa, Ethiopia.; ^4^UNICEF Nepal Country Office, Kathmandu, Nepal.; ^5^UNICEF Yemen Country office, Sana’a, Yemen.; ^6^The World Bank Group, Abidjan, Ivory Coast.; ^7^UNICEF Field office Benghazi, Benghazi, Libya.; ^8^UNICEF Regional Office West and Central Africa, Dakar, Senegal.; ^9^SingHealth Duke-NUS Global Health Institute, Singapore, Singapore.; ^10^University of Ottawa, Ottawa, ON, Canada.; ^11^UNICEF, New York City, NY, USA.

**Keywords:** Resilience, Monitoring and Evaluation, Health Systems, Community

## Abstract

**Background:**

The best approach for defining and measuring community healthcare (CHC) resilience in times of crisis remains elusive. We aimed to synthesise definitions and indicators of resilience from countries who had recently undergone shocks (ie, outbreaks and natural disasters).

**Methods:**

We purposively selected four countries that had recently or were currently experiencing a shock: Nepal, Ethiopia, Sierra Leone, and Liberia. Focus group discussions (FGDs) and key informant interviews (KIIs) were conducted with participants at the community, facility, district, sub-national, national, and international levels. Interviews and discussions were translated and transcribed verbatim. Data were open coded in ATLAS.ti using a grounded theory approach and were thematically collated to a pre-specified framework.

**Results:**

A total of 486 people participated in the study (n=378 community members, n=108 non-community members). Emergent themes defining CHC resilience included: the importance of communities, health system characteristics, learning from shocks, preventing and preparing for shocks, and considerations for sustainability and intersectoral engagement. Participants identified 193 potential indicators for measuring resilience, which fell into the domains of: (1) preparedness, (2) response and recovery, (3) communities, (4) health systems, and (5) intersectoral engagement.

**Conclusion:**

Despite varying definitions and understanding of the concept of resilience, community-centred responses to shocks were key in building resilience. Further insight is needed into how the definitions and indicators identified in this study compare to other shocks and contexts and can be used to further our understanding of health system resilience. Metrics and definitions could assist policy-makers, researchers, and practitioners in evaluating the readiness of systems to respond to shocks and to allow comparability across health systems. We must build health systems that can continue to function and ensure quality, equity, community-focused care, and engagement, regardless of the pressures put upon them and ensure they are linked to strong primary healthcare.

## Background

**Key Messages**

**Implications for policy makers**

- The term “resilience” has garnered renewed interest in the context of the global COVID-19 outbreak. Our analysis from another infectious disease outbreak (Ebola disease virus) and from natural disasters offers an important contribution for comparative analysis.
- This research could help countries to reconsider how resilience in community healthcare (CHC) is discussed, operationalized and understood at multiple levels throughout the health system. This understanding could assist in the identification of metrics or goals for community-based health systems to monitor and maintain their resilience on an ongoing basis.
- This research will help decision-makers plan and prepare their health systems (at all jurisdictional levels, ranging from local to regional to national) for emerging and future disruptions and/or shocks.

**Implications for the public**

This research could benefit the public by highlighting the importance of communities in the building of health system resilience. We offer various perspectives on the discourse around resilience building from multiple countries that have experienced shocks. We also offer perspectives from countries on which indicators to measure. These insights could help to inform resource allocation and promote decentralized crisis preparedness that places communities at the centre of the response. There are further implications for the importance of ensuring that primary healthcare-based health systems are supported to respond to multiple threats with a focus on the communities. Additionally, our research highlights the need for more community voices and community involvement in the discourse around what is resilience and how it can be measured.

Building resilience in health systems is imperative as health systems confront multiple, converging shocks with limited resources.^1^ “Shocks” can include sudden and severe events (eg, pandemic, natural disaster, armed conflict) as well as chronic stresses (eg, structural and political instability, ongoing staff shortages), including acute events that can become chronic problems.^2^ Maternal, newborn, and child health (MNCH) services are particularly vulnerable to disruption during shocks.^3,4^ In many low- and middle-income countries (LMICs) with recent outbreaks of Ebola virus disease, Zika, and COVID-19, progress that had been made toward improving MNCH indicators was halted or reversed (eg, family planning service utilization, antenatal health coverage, rate of institutional deliveries, child immunisation uptake).^4^ The global COVID-19 outbreak has reminded the world that country income status and whether a health system is well-resourced or strong are not synonymous with resilience in the face of disruption or shock. In the last two years, the contributions of communities to resilience have become a central focus worldwide as countries attempt to (re)build resilient health systems.

 Community healthcare (CHC) with a strong network of community health workers (CHWs) play an important role in building resilience, and are often the entry point to primary healthcare for community members.^5,6^ When crises or shocks occur it is often community-based healthcare settings that continue to provide basic health services. For example, during the Ebola virus disease response in Liberia, the availability of community-based healthcare ensured that essential child health services continued when facility-based care was compromised.^5^ In Nepal, following the earthquake in 2015, female community health volunteers provided the first wave of assistance prior to the arrival of aid from government or international relief agencies.^7^ During the COVID-19 response in South-East Asia, CHWs expanded their roles, conducted surveillance, and facilitated the continuation of essential health services.^8^ However, despite evidence of communities leading the way in responding to shocks in LMICs, the majority of support and resources have focused on emergency and facility-based services.^9^ There remains a need for better understanding of the factors that contribute to community-based health system resilience and what resilience truly means in order to strengthen this resilience around the world.^2,9-15^

 Health systems resilience has been defined in a variety of ways, which has challenged its utility.^16^ Kruk et al define resilience as “the capacity of health actors, institutions, and populations to prepare for and effectively respond to crises; maintain core functions when a crisis hits; and, informed by lessons learned during the crisis, reorganise if conditions require it.”^17^ Resilience is often conceptualised as an emergent property of health systems resulting from the dynamic and interconnected nature of complex systems.^14,18^ This systems orientation, as well as its emphasis on strengths, resources, and capacities rather than vulnerabilities and risks, is unique to a resilience-based approach.^19^

 However, there is a risk that the term resilience encourages unrealistic expectations for already disadvantaged communities providing actions requiring significant investment from local governments and international development actors.^20^ Some consider the resilience paradigm to be a form of neoliberal governmentality in which the conditions leading to crises are considered inevitable rather than shaped by political forces, thus placing the responsibility on individuals and communities to “bounce back” from shocks.^21^ Furthermore, the concept of “bouncing back” ignores the possibility that pre-shock, many health systems are chronically weak and perpetuate social inequalities.^22,23^ Some argue that health systems should strive for transformative resilience, or “transilience,” and “bounce forward” to avoid returning to a deficient status quo.^22^ Others emphasise the need to consider power relations and governance, and discuss the advantages of framing health system resilience as an ability rather than an outcome of a health system.^24^ In addition, the relationship between health system resilience and health system strengthening (HSS) remains ill-defined. Resilience has been described both as an outcome of a strong health system and a necessary component of it,^14,25,26^ while others use the terms interchangeably.^12,23^

 With the concept of resilience currently under debate, and given the renewed focus on rebuilding health systems in the aftermath of the COVID-19 pandemic, more work is needed to translate the concept of resilience into specific capacities and capabilities.^2,11,27^ Historically, the complexity of the concept of resilience has limited efforts to define measurable indicators of resilience.^11^ Thus, existing literature has been dominated by attempts to describe the general attributes of resilience rather than specific health system capacities.^27^ Moreover, many have highlighted the difficulty of applying standardized indicators in diverse contexts and settings that are experiencing varying types of shocks.^15,18,27,28^ In addition, many proposed indicators for measuring and assessing health system resilience lack an emphasis on CHC and direct input from countries that experience shocks.^15,18,28-30^ To overcome these challenges, some have suggested that benchmarks for each indicator should be set within each country.^15,18,27,28^ Others have focused on specific aspects of health system resilience, such as service utilisation changes during shock, but this may come at the expense of a comprehensive definition of resilience.^11^ Lastly, while the importance of gathering input from communities has been recognized in previous efforts to define resilience,^18,28,29^ few have emphasized resilience at the level of CHC.

###  Objective

 To address these gaps, this study sought to describe how countries that had recently experienced or were currently experiencing a shock define resilience in CHC and to summarise their recommendations for measuring resilience in CHC in low-resource settings, particularly in the context of MNCH.

###  Setting

 We conducted the study in four countries that had recently experienced or were currently experiencing shocks in 2015–2016: Nepal, Ethiopia, Liberia, and Sierra Leone. These represented natural disasters or infectious disease epidemics that had diverse population-level outcomes and health systems capacities to mitigate them. As seen in Table 1, the study represented diverse geographies and varying shocks.

**Table 1:** Four Countries Studied, the Shock, and Impact

**Country/Year(s)**	**Shock**	**Impact**
Nepal (2015)	7.8 magnitude earthquake followed by aftershocks	- Almost 9000 people dead - 2.8 million people displaced - 1200 health facilities destroyed
Ethiopia (2015-2016 and ongoing)	El Nino drought ongoing	- 80% loss of harvest leaving 8 million people in need of food assistance - Chronic malnutrition - Population migration - Spread of waterborne infections such as cholera
Sierra Leone (2014-2016)	West African Ebola Outbreak	- 3956 deaths - 14 124 cases
Liberia (2014-2016)	West African Ebola Outbreak	- 4809 deaths - 10 675 cases

 Nepal experienced an acute shock in 2015 in the form of a 7.8 magnitude earthquake that was followed by a series of aftershocks, killing almost 9000 people and displacing 2.8 million.^31^ More than 1200 health facilities were destroyed or damaged.^32^ In Ethiopia, the El Niño drought of 2015–2016 led to chronic malnutrition, population migration, and the spread of water-borne infections such as cholera. It resulted in an estimated loss of 80% of the harvest, leaving 8 million people in need of food assistance across the country.^33^ The drought has continued for several years thereafter and has been compounded by conflict. The West African Ebola outbreak of 2014–2016 resulted in the deaths of 4809 and 10 675 infections in Liberia and 3956 deaths and 14 124 cases in Sierra Leone.^34^

## Methods

###  Data Collection

 As part of a larger four-country study on building CHC resilience, we used qualitative methodologies to meet our research objectives. Key informant interviews (KIIs) and focus group discussions (FGDs) were conducted between January and October 2016. FGDs were chosen to elucidate shared knowledge of understanding of resilience among community level participants while KIIs were chosen to gain a deeper understanding of resilience. Initial interviewees were recruited by the United Nations International Children’s Emergency Fund (UNICEF) country-level staff using purposive sampling. Subsequent participants were identified by snowball sampling techniques until participants from across the health system had been reached. All participants were provided information sheets about the study, which was also verbally explained. All participants provided written informed consent to participate in the study and thumbprints were collected in lieu of signatures if respondent literacy was low. Data collection was informed by semi-structured interview guides based on country inputs and current literature and were tailored to participants’ roles in the health system. Participants who were categorised as “community participants” included community members, community or facility-based health workers, and members of community organizations. Community participants were asked if they had heard of the term resilience and if yes, asked what it meant and how we would measure it. Interviews at the community levels were conducted in English, Nepali, Afsomali, Amharic, Tigrinya, Kreyol, Mandingo, Kpelle, Temene, or Krio. If the interview was not conducted in English, simultaneous translation by a trained health worker was done to allow for probes and interaction between the participants and researcher. Data collection with non-community participants (ie, district, regional, national, and international level participants from governmental and non-governmental organizations) was conducted in English. Non-community participants were asked specific open-ended questions about how to define building resilience in the context of community-based health systems, whether they thought resilience was different from HSS, and how to measure resilience in CHC in their contexts. FGDs lasted up to 2 hours and KIIs were between 20 and 40 minutes long. All interviews were audio recorded with permission, transcribed verbatim, and translated to English if necessary. Translated transcripts were often verified with research assistants who facilitated the translation, but were not verified with participants due to logistical constraints.

###  Analysis

 English versions of transcripts were analysed using thematic content analysis based in a grounded theory approach. First level deductive coding was done in ATLAS.ti based on identifying definitions, measurements, and differences between HSS and resilience. Definitions and discourse on HSS were open coded using a grounded theory approach. The grounded theory approach was selected because of its strength in identifying both the interconnectedness of the data and areas of conflict or contradiction. Codes related to measurements were then applied to the five domains of the Kruk health system resilience index^18^ which included a priori themes of aware (tracks population health threats, maps system strengths and weaknesses, knows available resources), diverse (addresses a range of health problems, provides quality services to meet the populations needs), self-regulating (isolates health threats, minimizes disruption to essential services, can access reverse capacity), integrated (coordinates between governments, global and private actors, works across sectors, involves communities), and adaptive (transforms operations to improve function, acts on evidence and feedback, encourages flexible responses to fit the situation). The Kruk framework was chosen because it was the most comprehensive health system-focused resilience framework that also described sample indicators. Any discrepancies in the application of the framework were discussed with co-authors. We inductively coded the indicators further to identify emerging themes. Data were also compared between community and non-community perspectives as well as between shock types and countries in order to identify trends or differences in participant responses.

## Results

###  Participants

 Across the four countries, a total of 52 FGDs and 78 KIIs were conducted (Figure and Table 2). We had a total of 486 participants (378 community and 108 non-community participants) (Table 3). Of the 486 participants, those from Sierra Leone comprised the largest group at 37% (n = 181) of the participants followed by Liberia at 28% (n=134), Ethiopia at 19% (n = 94), and Nepal at 16% (n = 77). These participants represented 12 distinct geographies (counties, districts, and regions) in the 4 countries.

**Figure:**
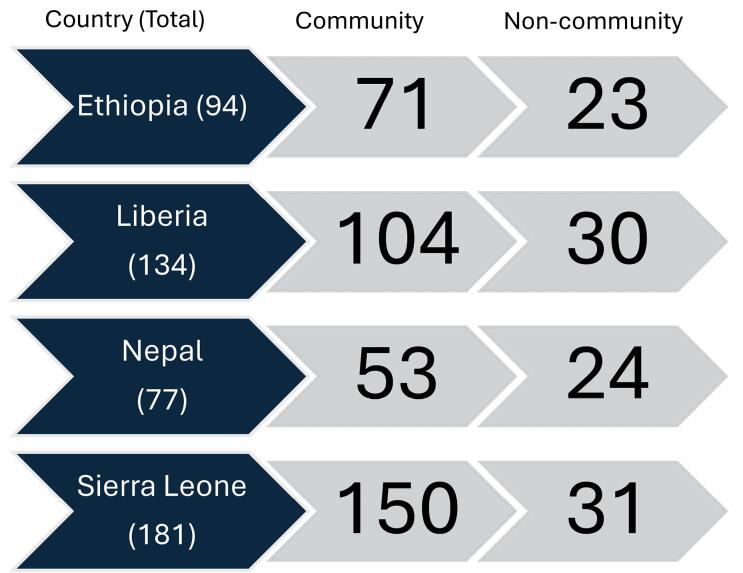
Participants by Country (n = 486).

**Table 2:** Numbers of Focus Group Discussions and Key Informant Interviews Per Country

**Country**	**FGDs**	**KIIs**
Ethiopia	11	17
Liberia	15	22
Nepal	6	22
Sierra Leone	20	17
**Total**	**52**	**78**

Abbreviations: FGDs, focus group discussions; KIIs, key informant interviews.

**Table 3:** Participants in 4 Country Study: Liberia, Sierra Leone, Ethiopia, and Nepal (n = 486)

**Community Participants (n = 378)**	**n**	**% Of Community Participants**
Ebola virus disease survivors	22	6%
Community leaders	121	32%
Local NGO/CBO	13	3%
Women groups	101	27%
Youth groups	4	1%
CHWs	71	19%
Healthcare workers	46	12%
**Non-community Participants (n = 108)**	**n**	**% Of Non-community Participants**
District/County	31	29%
Ministry of Health representatives	14	13%
UNICEF	34	31%
Partners (bilateral, multilateral, iNGOs)	29	27%
**Total**	**486**	

Abbreviations: NGO, non-governmental organisation; CBO, community-based organisation; CHWs, community health workers; iNGOs, international non-governmental organisations; UNICEF, the United Nations International Children’s Emergency Fund.

###  Defining Community Healthcare Resilience

 Key themes included the importance of community, health system properties (ie, being strong, adaptive, absorptive, coping, or bouncing back), learning from shocks, preventing and being prepared for shocks, and elements of sustainability and intersectoral engagement.

####  Community

 The most frequently discussed theme from the definitions of resilience was the centrality of communities in building resilience. Many definitions of resilience included the consideration of the community in health systems and community ownership of the response to a shock. Many participants described CHC resilience as conditional upon how communities were engaged, aware, trained, prepared, and able to use their own resources during shocks:

 “*Resilience is the ability for communities to be able to respond to shocks or to changes that they are experiencing often due to emergencies and disasters—they are able to cope and are adaptable” *(UNICEF Country Office, Nepal).

 “*The resilience in a system involves the engagement of the community so the health system can be very strong up to the lowest level from the health-seeking behaviour to prevention and response mechanisms. Health systems are strong, but the resiliencies add the community level” *(Federal Ministry of Health, Ethiopia).

 “I*f something happens the system must stand as [it was] before the events. If that system exists, that’s a resilient system. That might be in the health centre or the community level. So mainly in the community, urgency of mobilisation of those supportive hands to make available the necessary medical equipment or daily requirements. So, for that they must have training how to be safe by themselves first, then to provide the same thing to others” *(Ministry of Health, Nepal).

 “*We can say the health system is resilient if the community can name/know the health extension [community health] packages and have awareness about it” *(CHW, Ethiopia).

 Participants frequently described properties of a resilient CHC in terms of an ability to resist, bounce back, absorb, adapt, and cope with an emphasis on the community level. Many participants highlighted that resilience encompassed being able to respond to a variety of shocks, both known and unknown:

 “*Resilience means the way either the health system or the community can withstand any kind of problem without disruption—can cope and manage that problem with his own resources and any available resource on the ground” *(UNICEF Country Office, Ethiopia).

 “*[Resilience is] a health system that can resist man made and…natural disasters such as climate change, war, and some epidemics like scabies…” *(Federal Ministry of Health, Ethiopia).

 “*[Resilience is] the ability of a community or system to cope with the unexpected…whether it’s a natural disaster, or a war, or like an unusual situation” *(Ministry of Health, Nepal).

 Maintaining critical functions during a shock while responding to the new needs created from the shock was an important theme described in defining resilience. Many included an element of timeliness or speed in the response; especially providing high quality, essential services:

 “*If a health system is resilient, I would consider that health system strong. A resilient health system would be one that would be able to respond to an epidemic and at the same time provide routine health services. It will add a lot of strength, quality, and sustainability to the health system” *(Community Health Officer, Liberia).

 “*The health system should be resilient simply means the health system should be swift to provide and care for lives” *(Civil Society Member, Liberia).

 “*If the system is just capable of absorbing shock, managing shock, and then recovering from it the quickest possible and go forward to be able to deliver” *(International Partner, Nepal).

 “*[Resilience is] how soon a health system or health facility can respond back to normal…providing essential services as before [including] service delivery at the point of care and supply as well as the management of health services” *(UNICEF Country Office, Nepal).

 Many participants felt this was most important at the community level, but the health system overall also needed to be strong in order to be resilient.

####  Learning From Shocks and Building Back Better

 Some participants highlighted the need for health systems to be able to learn from the experience of shocks and evolve. Others indicated an imperative to build back better than before the shock, regardless of the initial strength of the health system:

 “*The part about resilience that we are trying to focus on is the way evidence can be more useful, is the way the system itself can adapt over time, so that it can learn from what it is doing right and what it is doing wrong and be able to grow stronger over time” *(Ministry of Health, Sierra Leone).

 “*[Resilience] adds that a health system can evolve to meet the needs over time regardless of how weak it is” *(Sierra Leone, Community Leader).

 “*Whatever we build back should be much better than what we did in the past—that is resilience. So the other aspect is the coping capacity of the community people so that they do not need to rely on external factor. So based on their own coping mechanism and capacity, they can build back better” *(UNICEF Country Office, Nepal).

####  Prevention and Preparedness

 In defining resilience, many spoke of the ability of a health system to prevent the shock if possible and to be prepared to address the effects of the shock on the health system and beyond. This included the use of early warning systems, and capacity building in communities, governments, and facilities. In their definitions of resilience, participants also described the ability for the health system to be prepared or cope with unknown threats as key to resilience:

 “*Resilience is just a system that is well prepared and ready to respond and it’s flexible to address problems whenever they occur so it’s something which is ready for change to really bring change to address problems” *(Implementing partner, Ethiopia).

 “*To build up the capacity of everybody—the communities, the health facilities, the government; we should be able to prepare ourselves early and respond in time in the event of any future outbreak not only related to the disease outbreak but also other non-heath outbreak. How our Ministry of Health and others are identifying the early warning systems” *(District manager, Sierra Leone).

####  Sustainability and Intersectoral Engagement

 Sustainability and intersectoral engagement were central to participants’ definitions of resilience. In many definitions, sustainability was a defining property of resilience, often emphasizing the self-reliance of communities as an important component of sustainability. Many participants noted that resilience in health systems goes beyond just the health sector. Water, agriculture, and education sectors were frequently described as important to CHC resilience. The engagement of all sectors in a cohesive effort to build resilience was often described:

 “*Being sustainable and self-reliant is resilience in a health system. We are looking at sustainability, something that is continuous and…is improved over time. For [those of] us that have worked with communities and also health systems, we see that health cuts across everything. Even if you are doing agriculture, it has a component of health; even water needs, sanitation, environmental needs—they are different aspects of the health system. So the system should look at all those areas to be resilient” *(Member of community-based organisation, Sierra Leone).

 “*We are talking about building the capacity of community or enhancing their capability to be able to withstand shocks and emergencies within the community and to help them regain back the previous status with regards to livelihoods, properties, and going on with their social and economic life including availability of healthcare, education, and social determinants of health” *(UNICEF Country Office, Ethiopia).

###  Comparing Health System Resilience to Health System Strengthening 

 Non-community participants held varying perspectives on whether there was a difference between a resilient or strong CHC and whether the resilience discourse added to the HSS discourse. Participants from regional, national, and international levels in all four countries held varied views on whether resilience and HSS were different. Many felt that strong health systems should inherently be able to handle anything (ie, be resilient) and therefore the terms were equivalent. Nonetheless, many also believed that resilience either added to the HSS discourse or that resilience and HSS were interrelated.

 The most commonly reported attribute of what resilience adds to HSS was regarding the capacity to withstand shocks and bounce back (including disasters or emergencies) as described in the following quote:

 “*A health system that is able to withstand the stresses of epidemics and various diseases, and be able to respond appropriately and remain intact, is what I consider resilient” *(CHW, Liberia).

 While HSS was considered to be a component of the routine or non-emergency functions of a health system, preparation or protection from shocks was highlighted by some participants as a component of resilience, and some felt resilience also included timely and efficient responses to shocks as seen in the following quote:

 “*Resilience is timely action to result in less death and hazard at every level of the health system” *(International Health manager, Nepal).

 Many felt resilience added the ability to detect and respond to shocks while maintaining core functions and without collapsing. Some felt resilience added the ability for health systems to cope, be flexible and adaptable, and focus on recovery. Some participants also felt resilience added considerations for resource mobilisation, engagement from the global community, and health system self-sufficiency.

 Outside the context of shocks, some participants felt resilience added a dimension of time to the HSS discourse, with some suggesting resilience developed over time while others felt it occurred only during shocks. This was also seen with HSS, where some participants described it as temporary, and others described it as progressive or long-term. Some participants also felt that resilience included elements of intersectoral collaboration, including both linking the health sector with social protection and looking for HSS beyond the health sector as described in the following quote:

 “*It is not only the health system [that] can be resilient enough to the respond to the public health emergency- community plus all other sectors are needed. Resilience is broader than even the health system” *(Federal Ministry of Health Manager, Ethiopia).

 A few participants also felt that resilience added considerations for communities (specifically engagement, participation, and trust), marginalised populations, social determinants of health, and sustainability.

 For those who felt resilience and HSS were interrelated, some described resilience as one element of HSS, or embedded within HSS, or a measurement of HSS. Others described HSS as a precursor, determinant, or product of resilience.

###  Measuring Resilience 

 Participants identified 193 potential indicators to measure resilience in CHC that fell into the broad categories of preparedness, response and recovery, communities, health systems, and intersectoral engagement(Table 4).

**Table 4:** Suggested Indicators to Measure Resilience in Community Healthcare Settings

**Theme**	**Sub-themes**	**Suggested indicator**
**Preparedness **		
	Planning	Presence of updated emergency plans in districts, health facilities, and communities Availability of a coordinated master disaster plan How coordinated plans are across sectors and stakeholders A triage plan of who to call when a shock occurs Plan if backup/stockpiled medication or supplies are destroyed District level planning
	Training	Frequency of disaster drills in the lowest levels of the health system People (including the communities) trained in shock or emergency response and disaster management
	Pre-positioned resources	Shock resistant infrastructure (eg, buildings and supply chains) and supplies (eg, vaccines and medications) at the community level Ear-marked resources that are easily deployed during a shock Investment of limited resources to equip health systems to be responsive
**Response and Recovery**		
	Mortality and morbidity related to shocks	Death and injuries from the shock (eg, mortality, numbers of amputations, fractures) Outbreaks or communicable disease emergence and their prevention (eg, cholera/water-borne pathogen outbreaks, morbidity and mortality resulting from outbreaks, diarrhoea caseload) Malnutrition (eg, severe acute malnutrition in children, newly acutely malnourished kids in shock affected areas, changes in rates of stunting, nutritional status of communities, growth monitoring)
	Timeliness of the response	Time-gap before resources are mobilised after a shock, services are resumed Rapidness of government response without international assistance Rapidness of the government response if another shock occurred Prompt restoration of health services Population that has access to food, shelter, and water 24 hours after a shock Ability of a health system to triage victims immediately after a shock (eg, triaging and transporting injured patients)
	*Recovery*	Using lessons learned from a shock and putting them into guidelines Use of community or local resources to rebuild or resume health services
**Communities**		
	*Awareness and strength of communities*	How proactive a community is to manage a shock How aware communities are of potential shocks Whether and how by-laws are enforced during a shock Strength of the networks at the community level Governance of networks at community level Support and supplies available to communities Defined responsibilities in communities Comparison of those communities impacted by shock with those not
	Link between communities and their health systems	Strength of referral systems from the community Effectiveness of CHWs linking communities and the health system Whether and how communities are engaged as part of the health system (eg, attendance of monthly meetings at facilities by community members, opportunities for communities to identify and remove health system bottlenecks)
**Health Systems**		
	Health service delivery and quality	Adaptability of the health system as disease burdens change Accessibility and equity of the health system to all populations (eg, how health systems deliver in remote communities) Uninterrupted health service provision, restoration of health services Health service utilisation in facilities and communities (eg, patients seen at the facility per day, health service utilisation per population, availability of essential health packages, numbers of children referred from communities who attended facilities, if communities have access to health services and whether they are using them) Comparing service delivery indicators before, during and after a shock and/or in shock-affected areas compared to non-shock affected areas Appropriateness of service availability at various levels in the health system Quality of care (eg, quality of services available at health facilities, whether workload can be managed in the facilities, effectiveness of management in the health facilities) *MNCH Services* Access to antenatal care (compared before, during, and after a shock) Availability of safe motherhood services during a shock (eg, prevention of maternal sepsis, obstetric haemorrhage/severe bleeding, venous thromboembolism/blood clots, and severe hypertension in pregnancy, safe deliveries/institutional deliveries) Integrated management of childhood illnesses Childhood immunisation (eg, number of children fully vaccinated in facilities and communities, immunisation coverage and drop out, continuation of immunisation during a shock) Accessibility of family planning
	Health workers	Numbers of trained health workers (eg, numbers: of CHWs, facility-based health workers, professional health workers, support staff) Numbers of health workers who have resumed their roles after a shock Distribution, motivation, and capacity of trained staff to respond to a shock Compare the motivation of health workers in shock area to non-shock area (eg, timeliness of payments) Training for health workers is monitored and followed-up
	Infrastructure and supply	If and how long supplies and logistics are disrupted after/during a shock Numbers of health facilities Availability of essential equipment Robustness of supply chains (eg, stock out of medication and basic commodities)
	Monitoring and evaluation or surveillance	Data availability and community level and whether it is monitored and acted upon during a shock Data and records that are accessible during a shock Number of times the primary healthcare level or district level acted when there the surveillance indicated there was a potential threat Whether surveillance systems can detect a shock or threat of a shock Community-based monitoring and social accountability tool/score cards Completeness/timeliness of community-based reports
**Intersectoral Engagement**		
		How information flows across sectors Education (eg, management of shock in school, school attendance/drop out, number of teachers trained in shock preparedness and management) Water, hygiene, and sanitation (eg, availability of clean, safe drinking water and a family’s knowledge of appropriate water use, hygiene and sanitation, solid and liquid waste removal) Agriculture (eg, availability of food, productivity including technology)

Abbreviations: CHWs, community health workers; MNCH, maternal, newborn, and child health.

####  Preparedness

 The most commonly suggested indicators to measure resilience in CHC in the context of preparedness were related to the presence and adequacy of emergency plans. Updated emergency plans that were available at the district level, in health facilities, and in communities were frequently recommended, as were plans that were coordinated across sectors and stakeholders. A plan for who to call and how to triage during a shock was also recommended. One participant suggested measuring resilience by whether a plan existed in case stockpiled medication or supplies are destroyed. Planning at the district level and within communities were often described as measurements of resilience. The presence of trained people, including the frequency of disaster drills in the lowest levels of the health system and numbers of people trained in disaster management or emergency response, especially within communities were other often discussed metrics.

 Participants often described the existence of pre-positioned resources as a measure of resilience. This included infrastructure (eg, buildings, supply chains) and stockpiles (eg, medical supplies, vaccines, medications) and was especially important within communities. Many participants spoke of having ear-marked resources that could be easily deployed at the time of shock as a measure of resilience. One participant recommended measuring how scarce resources were invested to equip health systems to be responsive to a shock.

####  Response and Recovery

 Many participants described measuring population impacts of a shock and how to prevent these impacts. These included measuring morbidity, mortality, and injuries (fractures and amputations) from acute shocks, as well as cases of and deaths from communicable disease outbreaks (both those that are shocks themselves and those that emerge in the wake of other shocks). The nutritional status of the population (eg, severe acute malnutrition in children, acute malnutrition in shock impacted areas, changes in rates of stunting, growth monitoring, nutritional status of communities) was also a suggested indicator because in order to be successful multiple sectors would need to function together.

 Suggested indicators related to the response were often time-bound (eg, within 8, 24, 48, and 72 hours) or the indicator itself was about the “time gap” between the shock and the restoration of essential services or a response to urgent needs (eg, time gap before resource mobilisation, speed of the government response without international assistance, speed of the government response if another shock occurred). Many suggested indicators focused on the use of local or government resources during the response and recovery phase, including the prompt restoration of health services or the availability of essential services immediately after a shock (eg, water, shelter, food). Some participants suggested the use of community resources to rebuild after a shock would be an indicator of resilience. Triaging and transporting victims of a shock during the response was also mentioned frequently as a potential indicator. Lastly, participants recommended measuring whether lessons learned from a shock were put into guidelines.

####  Communities

 Many of the suggested indicators situated communities as central actors in the health system and directly measured resilience in terms of communities’ participation, linkage, engagement, and the strength of their networks. Many participants suggested measuring how aware and proactive communities were of potential shocks and whether they were able to enforce by-laws during a shock. Governance and network strength measurement in communities were proposed as indicators based on whether communities had defined responsibilities. Some suggested comparing communities impacted with shocks to those not impacted by a shock to identify differences between them that could be indicators of resilience.

 Many community indicators were related to the effectiveness of linking communities to their health systems. This included the strength of referral networks from communities to facilities and the link between CHWs, communities and their health systems. Opportunities for communities to participate in their health systems (eg, monthly health meetings at facilities and the attendance by community members, opportunities for community members to identify and remove health system bottlenecks, and whether and how communities are engaged) were often proposed indicators of resilience across the countries and perspectives.

####  Health Systems 

 Indicators related to health systems fell into broad categories of health service delivery and quality, health workers, infrastructure and supply chains, and monitoring and evaluation, including surveillance. Participants suggested measuring how adaptable a health system is when the disease burden changes, as well as the accessibility and equity of a health system (eg, how health systems deliver in remote communities) as indicators of resilience.

 Health service utilization in communities and facilities (eg, patients seen at the facility per day, availability of essential health packages, number of children referred from communities who attended facilities, if communities have access to health services and whether they are using them) were the most frequently described metrics related to service delivery. Many participants also recommended comparing service delivery indicators before, during and after a shock and/or in shock-affected areas compared to non-shock affected areas. One participant recommended examining the appropriateness of service availability at various levels in the health system to measure resilience. A few participants described measuring the quality of care of health services provided in the facilities as a measure of resilience and this included examining the effectiveness of management at facilities and whether they could cope with the workload.

 Many indicators for measuring resilience with respect to health service delivery were related to MNCH. These included measuring access to antenatal care (before, during, and after a shock) and safe motherhood (eg, institutional deliveries or safe deliveries in communities during shocks, the prevention of maternal sepsis, obstetric haemorrhage, venous thromboembolism, and severe hypertension in pregnancy). Some participants recommended measuring access to integrated management of childhood illnesses in communities, especially regarding childhood immunisations (eg, number of children fully vaccinated in facilities and communities, immunisation coverage and drop out, continuation of immunisation during a shock). Lastly, one participant recommended measuring access to family planning.

 Participants also recommended indicators related to health workers to measure resilience. Numbers of health workers was the most frequently described indicator (eg, numbers of CHWs, facility-based health workers, professional health workers, support staff). Some participants recommended looking at the numbers of health workers who have resumed their roles after a shock as a measure of resilience. The distribution, motivation, and capacity of health workers to respond to shocks were frequently recommended indicators, especially comparing the motivation in shock and non-shock areas; this included timeliness of payments. Lastly, many participants recommended measuring the training and capacity building of health workers and whether training was followed up to see if it was successful.

 Indicators related to measuring infrastructure and supply chains were often recommended by participants. Numbers of health facilities, availability of equipment, and the existence of basic infrastructure to deliver healthcare were often discussed. This also included measuring the robustness of the supply chain, including the availability of medication and basic health commodities and how long supply chains were disrupted during a shock.

 Monitoring, evaluation, and surveillance indicators focused on the ability to detect and respond to a shock, emergency, or changing patterns of diseases. Many suggested indicators focused on robust information systems that could provide quick, accurate, and actionable data. At the community levels, the availability of completed and timely community-based reports as well as accurate data were proposed indicators.

####  Intersectoral Engagement

 Intersectoral engagement was a common theme reported by participants, and how information flowed across sectors was a suggested indicator. Many participants described education sector-related indicators to measure resilience, such as how the shocks are managed at schools, school attendance and drop out, and whether teachers are trained to prepare for the shock. Water, hygiene, and sanitation were often described as critical in measuring resilience, including the availability of clean, safe drinking water and a family’s knowledge of appropriate water, hygiene, and sanitation practices (eg, solid and liquid waste removal). Food and nutrition sector factors were also suggested as indicators, including the availability of food and agricultural productivity using technology inputs.

## Discussion and Conclusion

 Health system resilience has garnered renewed interest in the wake of the global COVID-19 outbreak and the emergence of monkeypox in non-endemic countries. These shocks have revealed cracks in health, social, political, economic, and food systems, exacerbating inequities within and between countries. In the context of (re)building resilient public health systems, new discourses have arisen around how acute stressors and chronic stressors impact resilience in health systems.^35^ While this debate continues, our research offers a framing for local CHC resilience measurement and solutions.

 Our analysis and proposed definitions and measurements from shocks to four national health systems make an important contribution by bringing perspectives directly from countries for comparative analysis for health systems. That community is at the core of every national health system—both as health service providers (ie, community-based health workers) and as health service users (ie, community members)—was evident throughout our findings. The capability to retain essential health services; adapt rapidly to changed and changing circumstances; and “bounce back” following shock were key areas where potential indicators were raised. Timeliness, intersectoral engagement, and sustainability were key themes that emerged throughout our discussions with participants.

 Responses to shocks that were centred around communities continued to be key in building resilience in CHC. Communities have been recognized as critical actors in building resilience, as discussed by Haldane and Morgan.^22^ We found the strength of the communities and their link to the primary healthcare focused health systems were key in building resilience, similar to recommendations from the World Health Organization’s (WHO’s) health system resilience indicators package.^36^ While 6 of the 64 recommended indicators in the WHO package focused on community engagement and participation, none of these indicators captured the strength and resilience of communities as a determinant of CHC. Participants described how active and engaged communities were in building resilience as essential elements of resilience in CHC, with much discussion on community ownership of the response and health system at large. CHWs continued to be the intersection between communities and their health system and provided essential health services during times of shocks.^5,37,38^ Bhandari and Alonge suggest metrics for measuring community resilience that should be incorporated in health system metrics and included documenting community ownership of the response, planning and participation of communities.^15^ Surveillance and monitoring at the community levels were also important, as was whether or not changes observed were acted upon- further highlighting the importance of the link between communities and their health systems. Therefore, investments in robust information systems at the community levels are also investments in resilience building.

 Many of the service delivery indicators included elements of returning to status-quo, with little discussion of improvement beyond what was present prior to the shock, although this improvement was present in the definitions of resilience provided by participants. However, definitions were not consistent as described elsewhere and without a common definition of resilience, the ability to translate discourse into practice remains limited.^2,11^

 Many of the service delivery indicators recommended by participants focused on MNCH. During the COVID-19 response, reproductive and maternal health were seen to be more resilient to changes, with mixed results for facility-based deliveries and clear declines in childhood immunisation.^39^

 Time was an important element discussed in the resilience of CHC, especially in relation to shocks. Many participants described indicators to measure CHC resilience that could be measured prior to a shock (preparedness), during, and/or after. Since the term “shocks” could encompass events that are relatively short or acute (eg, earthquake, coup d’état) as well as protracted or chronic events (eg, drought, COVID-19, financial or political shocks) or both simultaneously, it is important that indicators are routinely measured before, during, and after shocks to be able to detect changes to baseline. While before and after comparisons are often used to describe changes in health systems, we must examine the “before” with caution, as discussed by Haldane and Morgan.^22^ Disparities and inequities existed prior to the shock, meaning countries should not necessarily aim to “bounce back” but to also address inequities in population health that can be addressed within or are perpetuated by the health system. This is especially important in the context of learning health systems which have been identified as a key priority for LMICs to achieve greater self-reliance for their health systems.^40^ The rapid sharing of evidence from exemplary health systems and novel models of service delivery (eg, competencies for health workers to build people’s self-care) and evidence around how measuring resilience that can be implemented and institutionalised is needed. Early evidence from the COVID-19 pandemic suggests regions with learning health systems and experience of previous epidemics have been better able to respond to COVID-19.^41,42^

 Drawing from the diverse examples of this study, countries and communities should be encouraged to leverage potential influx of resources and use locally available resources to rebuild health systems in a way that they are more likely to be resilient and meet the needs of communities in normal times and during times of shock.

 Intersectoral engagement and sustainability were two themes that were prominent in participants’ discussions on resilience of CHC. As described by Meyer et al, it is important to address broader social determinants of health and understand the factors that prevented health systems from becoming resilient in the first place including structural, economical, and political barriers.^28^ Efforts need to continue to be made to converge on a common definition of resilience that also goes beyond traditional health system building blocks to actively engage communities, account for determinants of health across all sectors (eg, water, education, agriculture), and ensure investments are sustainable so that decentralised responses to shocks can continue over time.

 While debate remains on whether health system resilience could or should be measured, the term resilience remains more ubiquitous than ever, particularly in the aftermath of the global COVID-19 pandemic.^2,11,24^ Our participants felt it added value to the HSS discussion. However, further understanding is needed as to how to measure health systems strengthening and resilience, especially as the global community moves towards providing universal health coverage.^43^ In building health system resilience, metrics could assist policy-makers, researchers, and practitioners in evaluating the readiness of systems to respond to shocks and allow comparability across health systems, and many such metrics development exercises are well underway.^35^ With ever increasing direct and indirect health threats globally, the imperative to build health systems that provide quality, accessible, equitable, and community-focused health services able to function in the face of pressure continue to build our learnings and improve resilience in health systems.

###  Strengths and Limitations

 The strengths of this study are the large variety of participants across four countries at multiple levels of the health systems. Furthermore, participants had experienced shocks recently, limiting the risk of recall bias. That said, a potential limitation of our study is that as shocks may have continued, returned or been exacerbated by overlapping shocks (as in Ethiopia), participants’ conceptualisations of resilience or how they would measure it may have changed over time, highlighting the importance of health systems as learning health systems. Additionally, the term resilience was used in English for interviews and focus groups in order to obtain an unbiased definition. In cases, where participants were unfamiliar with the term, research assistants translated resilience based on the definition by Kruk et al.^17^ The interpretations and translations varied based on local languages potentially leading to biased interpretations. Lastly, few community members had heard of the term resilience and therefore the majority of this data comes from non-community members and may not reflect the priorities and needs of communities.

 In conclusion, despite varying definitions and understanding of the concept of resilience, community-centred responses to shocks were key in building resilience in CHC. Many suggested indicators included a time measurement and return to status-quo, and considerations for intersectoral engagement and sustainability were often discussed. Further insight is needed on how to quickly learn and implement findings in health systems. Metrics and definitions could assist policy-makers, researchers, and practitioners in evaluating the readiness of systems to respond to shocks and allow comparability across health systems. The importance of community participation in health systems and linking communities to strong primary healthcare-based health systems remains paramount. We must build health systems that ensure quality, equity, community-focused care, and engagement that can continue to function regardless of the pressures put upon it.

## Acknowledgements

 We gratefully acknowledge our research participants for sharing their valuable time, experiences and insights. We would also like to acknowledge the country office team members of UNICEF who assisted in coordinating the research. We would like to acknowledge the Rockefeller foundation for their support. We would like to acknowledge David Hipgrave for his insightful comments to strengthen the manuscript.

## Ethical issues

 Ethical approval was obtained from both the University of British Columbia’s Behavioural Research Ethics Board and individual country ethical review boards (Nepal Health Research Council, Ethiopian Public Health Institute’s Scientific and Ethical Review Committee, University of Liberia Institutional Review Board and Office of the Sierra Leone Ethics and Scientific Review Committee). All participants provided written informed consent.

## Conflict of interests

 Authors declare that they have no conflict of interests.
